# Combining New Non-Nucleoside Reverse Transcriptase Inhibitors (RTIs) with AZT Results in Strong Synergism against Multi-RTI-Resistant HIV-1 Strains

**DOI:** 10.3390/molecules23071599

**Published:** 2018-07-02

**Authors:** Fei Yu, Wen Li, Lili Wang, Yu Dai, Xin Lu, Qian Wang, Lan Xie, Shibo Jiang

**Affiliations:** 1 College of Life Sciences, Hebei Agricultural University, Baoding 071001, China; feiyu1110@hotmail.com (F.Y.); qwertydaiyu@163.com (Y.D.); luxin0409@163.com (X.L.); 2 Key Laboratory of Medical Molecular Virology of MOE/MOH, School of Basic Medical Sciences, Fudan University, 130 Dong An Rd., Xuhui District, Shanghai 200032, China; wenli3708@gmail.com(W.L.); wang_qian@fudan.edu.cn (Q.W.); 3 Research Center of Chinese Jujube, Hebei Agricultural University, Baoding 071001, China; lily850908@163.com; 4 Beijing Institute of Pharmacology & Toxicology, Beijing 100850, China; lanxie4@icloud.com; 5 Lindsley F. Kimball Research Institute, New York Blood Center, New York, NY 10065, USA

**Keywords:** HIV-1, HAART, NNRTIs, antiviral activity, combination, synergism

## Abstract

Reverse transcriptase inhibitors (RTIs), including nucleoside RTIs (NRTIs) and non-nucleoside RTIs (NNRTIs), are critical antiretroviral drugs for the treatment of human immunodeficiency virus (HIV) infection. Emergence of multi-RTI resistance calls for the development of more potent therapeutics or regimens against RTI-resistant strains. Here, we demonstrated that combining azidothymidine (AZT) with a new NNRTIs under development, diarylpyridine (DAPA)-2e, diarylanilin (DAAN)-14h, or DAAN-15h, resulted in strong synergism against infection by divergent HIV-1 strains, including those resistant to NRTIs and NNRTIs, suggesting the potential for developing these novel NNRTIs as salvage therapy for HIV/acquired immune deficiency syndrome (AIDS) patients.

## 1. Introduction

**Figure 1:**
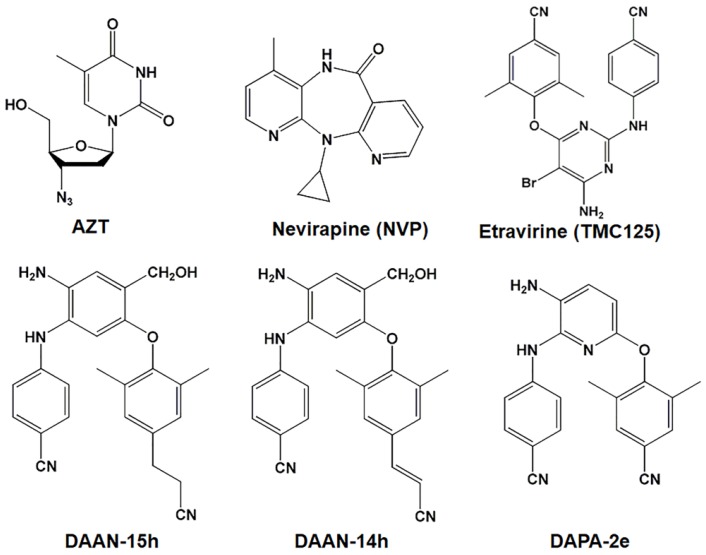
Chemical Structure of the nucleoside reverse transcriptase inhibitor (NRTI) azidothymidine (AZT) and five non-nucleoside reverse transcriptase inhibitors (NNRTIs), including Nevirapine (NVP), Etravirine (TMC125), diarylanilines (DAANs)-15 h, DAAN-14 h, and diarylpyridines (DAPA)-2e.

Reverse transcriptase (RT) is an important target for the development of anti-HIV-1 drugs (HIV: human immunodeficiency virus) because of its important role in the HIV-1 life cycle [1]. RT inhibitors (RTIs) include a variety of nucleoside and non-nucleoside reverse transcriptase inhibitors (NRTIs and NNRTIs) that inhibit the conversion of single-stranded viral RNA into double-stranded pro-viral DNA in the HIV-1 infection process [2]. These RTIs are key components of the highly active antiretroviral therapy (HAART) used in clinics [3,4]. However, the rapid emergence of multi-RTI resistance has led to the failure of patients to respond to the current HAART. Recently, Xie and colleagues have identified two classes of novel HIV-1 NNRTIs, diarylanilines (DAANs) and diarylpyridines (DAPAs) (see Figure 1), with extremely high anti-HIV efficacy and improved resistance profile [5,6,7,8]. As a further study, we combined new DAPA or DAAN-NNRTIs (i.e., DAPA-2e, DAAN-14h, and DAAN-15h) with azidothymidine (AZT) [9,10] to explore their potential synergistic antiviral effects against laboratory-adapted and primary as well as RTI-resistant HIV-1 strains. Meanwhile, NNRTI drugs nevirapine (NVP) [11] and etravirine (ETR or TMC125) [12] were used as controls because the synergy between AZT and NVP [13] or between AZT and ETR [14] have been previously reported. Herein, we reported their synergistic results of new DAPA or DAAN-NNRTIs/AZT combinations.

## 2. Results and Discussion

As shown in Table 1, all NNRTI/AZT combinations exhibited synergistic effects against infection by the laboratory-adapted HIV-1 strains IIIB (subtype X4) and Bal (subtype R5), and primary HIV-1 isolates 94US_33931N (subtype R5) and 93IN101 (subtype C, R5), with combination index (CI) in the range of 0.025 to 0.904. The DAAN-15h/AZT combination showed the strongest synergism against HIV-1 IIIB infection with a CI of 0.071, and dose reduction of DAAN-15h was about 44-fold, while that of AZT was about 21-fold. Combining AZT with the novel NNRTI DAPA-2e, DAAN-14h, or DAAN-15h, all exhibited strong synergism, which is comparable to that of the combination of AZT with the FDA-approved NNRTI drug TMC125 or NVP, suggesting that these new NNRTIs have the potential to be used for HIV/acquired immune deficiency syndrome (AIDS) patients who have failed to respond to the currently used NNRTIs.

Subsequently, we tested NNRTI/AZT combinations against AZT-resistant strains 964 and 629. When tested alone, the IC_50_ values of AZT against these two resistant strains were 15,178 and 41,109 nM, respectively, whereas those of NNRTIs tested alone were in the range of 0.6 to 34 nM. In the combinations, the IC_50_ values of AZT against these two resistant strains were in the range of 7 to 4797 nM, whereas those of NNRTIs ranged from 0.02 to 18 nM, with CI < 0.3 (see Table 1). In general, AZT combined with DAAN-14h, DAPA-2e, and DAAN-15h exhibited stronger synergism against the two resistant strains than the combination of AZT with TMC125 or NVP (see Table 1). For example, the CI values for combinations of AZT with new NNRTIs against AZT-resistant strains 964 and 629 were in the range of 0.003–0.040 and 0.109–0.237, respectively, while the CI values for combinations of AZT with the currently FDA-approved NNRTIs against AZT-resistant strains 629 and 629 ranged from 0.231 to 0.262 and from 0.292 to 0.423, respectively (see Table 1).

Results suggest that these new NNRTIs may be used to treat patients against HIV-1 mutants resistant to the currently available RTIs.

Finally, we investigated the cooperative effects of these new NNRTIs in combination with AZT against the multiple RTI-resistant strain RTMDR1. This strain contains mutations in RT amino acid residues 74V, 41L, 106A, and 215Y, rendering it resistant to many NRTIs and NNRTIs [15]. RTMDR1 appeared particularly resistant to NVP and AZT, with IC_50_ of 255 and 935 nM, respectively, when tested alone. On the other hand, this HIV-1 strain was relatively sensitive to TMC125, the recently approved NNRTI, as well as the new NNRTIs under development including DAPA-2e, DAAN-14h, and DAAN-15h in the range of 1.34–24.46 nM. All NNRTI/AZT combinations exhibited strong synergism against the multi-RTI-resistant strain RTMDR1, with CI ranging from 0.116 to 0.279 and dose reduction in the range of 7- to 18-fold for both NNRTIs and AZT (see Table 1). These findings suggest that combining NNRTIs with AZT leads to a strong synergism against infection by HIV-1 mutants resistant to both NRTIs and NNRTIs.

In summary, the new NNRTIs under development, DAPA-2e, DAAN-14h, and DAAN-15h, possess improved antiviral activity against HIV-1 strains, particularly those resistant to RTIs. Here we found that combining these new NNRTIs with AZT resulted in synergism, or strong synergism, against divergent laboratory-adapted and primary HIV-1 strains, as well as those resistant to NRTIs and NNRTIs. Therefore, these new NNRTIs can be further developed as new additions to the anti-HIV drug arsenal, and they can be effectively used as salvage therapy for HIV/AIDS patients who have failed to respond to currently available antiretroviral drugs or as anti-HIV microbicides for prevention of sexual HIV transmission. Nevertheless, pre-clinical studies on the in vivo efficacy and safety, including the long term toxicity, of these NNRTI/NRTI combinations and on the selection of HIV-1 drug mutants are warranted in order to establish the therapeutic potential of these drug combinations in clinic application. In addition, the combinations of these investigational NNRTIs with the modern, preferred NRTIs such as tenofovir (TDF/TAF) and FTC should also be tested in the future.

## 3. Materials and Methods

### 3.1. Reagents

CEMx174 5.25M7 cells, HIV-1 inhibitors AZT, TMC125, and NVP, laboratory-adapted HIV-1 strains IIIB and Bal, primary HIV-1 strains 94US_33931N and 93IN101, AZT-resistant strains 964 and 629, and the RTI-resistant strain RTMDR1 were all obtained from the National Institutes of Health AIDS Research and Reference Reagent Program. The small molecules NNRTI DAPA-2e, DAAN-14h, and DAAN-15h were synthesized as previously described [5,6,7,8].

### 3.2. Viral Infectivity Assay

The inhibitory activities of different drugs on infection by different HIV-1 strains were tested in CEMx174 5.25M7 cells by p24 assay as previously described [16,17,18]. Briefly, in the presence or absence of the tested inhibitors at graded concentrations, CEMx174 5.25M7 cells expressing CD4 and coreceptors CXCR4 and CCR5 were infected with a HIV-1 strain at 100 TCID_50_ (50% tissue culture infective dose). On the fourth day post-infection, culture supernatants were collected to test for p24 antigen by ELISA as previously described [19]. The ratio of NNRTI (DAAN or DAPA) to NRTI (AZT) in these combinations was determined based on their IC_50_ (concentration of an inhibitor achieving 50% inhibition of viral infection) values when tested alone. For example, the IC_50_ values of DAPA-2e (NNRTI) and AZT (NRTI) for inhibiting HIV-1 IIIB infection when tested alone are 99.21 and 39.31 nM, respectively (see Table 1). The ratio for DAPA-2e:AZT in the combination is 2.5 (=99.21/39.31), to make the concentrations of the NNRTI and NRTI in the combination with equal potency. The ratios for other NNRTIs to AZT were calculated in the same way. IC_50_ and CI (combination index) values were calculated using the CalcuSyn program [20,21,22]. The strength of synergism is indicated by the following CI values: <0.1: very strong synergism; 0.1–0.3: strong synergism; 0.3–0.7: synergism; 0.7–0.85: moderate synergism; 0.85–0.90: slight synergism.

## 4. Conclusions

In summary, our results suggest that these newly identified NNRTIs can be used in combination with NRTIs, such as AZT, as salvage therapy for HIV/AIDS patients who have failed to respond to currently available antiretroviral drugs, or as anti-HIV microbicides for prevention of sexual HIV transmission.

## Figures and Tables

**Table 1:** Combination index (CI) and dose reduction in inhibition of infection by the HIV-1 strains by combining NNRTIs and AZT.

**HIV-1 Strains (Tropism)**	**CI**	**DAPA-2e**			**AZT**		
		**IC_50_ (nM)**		**Dose Reduction (Fold)**	**IC_50_ (nM)**		**Dose Reduction (Fold)**
		**Alone**	**in Mixture**		**Alone**	**in Mixture**	
IIIB (X4)	0.134	99.21	3.05	32.50	39.31	4.07	9.66
Bal (R5)	0.364	70.50	8.42	8.38	34.47	8.42	4.10
94US_33931N (R5)	0.652	11.51	4.23	2.72	148.91	42.32	3.52
93IN101 (C, R5)	0.089	34.24	0.29	116.19	730.12	58.95	12.39
964 (R5/X4)	0.003	3.35	0.01	460.00	15,178.32	7.28	2083.61
629 (R5/X4)	0.156	34.49	2.37	14.52	41,109.61	3562.15	11.54
RTMDR1 (X4)	0.169	24.46	1.61	15.16	935.39	96.82	9.66
**HIV-1 Strains (Tropism)**	**CI**	**DAAN-14h**			**AZT**		
		**IC_50_ (nM)**		**Dose Reduction (Fold)**	**IC_50_ (nM)**		**Dose Reduction (Fold)**
		**Alone**	**in Mixture**		**Alone**	**in Mixture**	
IIIB (X4)	0.144	39.12	2.42	16.18	39.31	3.22	12.20
Bal (R5)	0.528	3.77	0.31	12.26	34.47	15.39	2.24
94US_33931N (R5)	0.904	1.65	0.71	2.33	148.91	70.74	2.11
93IN101 (C, R5)	0.141	1.55	0.07	22.09	730.12	70.25	10.39
964 (R5/X4)	0.023	0.62	0.01	54.04	15,178.32	69.23	219.26
629 (R5/X4)	0.109	13.87	0.84	16.55	41,109.61	2010.51	20.45
RTMDR1 (X4)	0.279	1.34	0.20	6.67	935.39	120.26	7.78
**HIV-1 Strains (Tropism)**	**CI**	**DAAN-15h**			**AZT**		
		**IC_50_ (nM)**		**Dose Reduction (Fold)**	**IC_50_ (nM)**		**Dose Reduction (Fold)**
		**Alone**	**in Mixture**		**Alone**	**in Mixture**	
IIIB (X4)	0.071	3.98	0.09	44.22	39.31	1.86	21.13
Bal (R5)	0.852	5.36	0.52	10.31	34.47	26.02	1.32
94US_33931N (R5)	0.063	0.47	0.02	20.72	148.91	2.27	65.65
93IN101 (C, R5)	0.095	0.60	0.02	27.72	730.12	43.21	16.90
964 (R5/X4)	0.040	0.74	0.02	32.03	15,178.32	139.03	109.17
629 (R5/X4)	0.237	16.57	2.00	8.29	41,109.61	4797.90	8.57
RTMDR1 (X4)	0.116	1.59	0.09	17.45	935.39	54.48	17.17
**HIV-1 Strains (Tropism)**	**CI**	**TMC125**			**AZT**		
		**IC_50_ (nM)**		**Dose Reduction (Fold)**	**IC_50_ (nM)**		**Dose Reduction (Fold)**
		**Alone**	**in Mixture**		**Alone**	**in Mixture**	
IIIB (X4)	0.179	0.89	0.08	10.66	39.31	3.35	11.73
Bal (R5)	0.883	3.20	1.93	1.66	34.47	9.65	3.57
94US_33931N (R5)	0.203	2.09	0.18	11.86	148.91	17.62	8.45
93IN101 (C, R5)	0.110	1.49	0.03	46.05	730.12	64.76	11.27
964 (R5/X4)	0.231	0.73	0.13	5.58	15,178.32	789.89	19.22
629 (R5/X4)	0.292	5.86	1.20	4.89	41,109.61	3599.14	11.42
RTMDR1 (X4)	0.194	1.24	0.17	7.21	935.39	51.74	18.08
**HIV-1 Strains (Tropism)**	**CI**	**NVP**			**AZT**		
		**IC_50_ (nM)**		**Dose Reduction (Fold)**	**IC_50_ (nM)**		**Dose Reduction (Fold)**
		**Alone**	**in Mixture**		**Alone**	**in Mixture**	
IIIB (X4)	0.199	11.74	1.47	8.01	39.31	2.93	13.41
Bal (R5)	0.892	307.91	50.25	6.13	34.47	25.12	1.37
94US_33931N (R5)	0.316	24.15	2.91	8.28	148.91	29.15	5.11
93IN101 (C, R5)	0.025	33.64	0.10	343.92	730.12	16.30	44.78
964 (R5/X4)	0.265	1.28	0.27	4.73	15,178.32	809.01	18.76
629 (R5/X4)	0.429	29.68	6.82	4.35	41,109.61	8832.58	5.02
RTMDR1 (X4)	0.132	255.16	18.55	13.75	935.39	55.66	16.81

Note: HIV = human immunodeficiency virus. IIIB and Bal are laboratory-adapted HIV-1 strains, 94US_33931N and 93IN101 are primary HIV-1 strains, 964 and 629 are AZT-resistant HIV-1 strains, and RTMDR1 is the multiple RTI-resistant HIV-1 strain. A CI of >1, 1, and <1 indicates antagonism, additive effect, and synergism, respectively. The strength of synergism is indicated by the following CI values: <0.1: very strong synergism; 0.1–0.3: strong synergism; 0.3–0.7: synergism; 0.7–0.85: moderate synergism; and 0.85–0.90: slight synergism. Dose reduction (fold) was calculated using the following formula: IC_50_ value of an inhibitor tested alone/the IC_50_ value of the same inhibitor tested in combination with another inhibitor.
